# Genome-Wide Analysis on Driver and Passenger RNA Editing Sites Suggests an Underestimation of Adaptive Signals in Insects

**DOI:** 10.3390/genes14101951

**Published:** 2023-10-17

**Authors:** Yuchen Zhang, Yuange Duan

**Affiliations:** MOA Key Lab of Pest Monitoring and Green Management, Department of Entomology, College of Plant Protection, China Agricultural University, Beijing 100193, China; zhangyc@cau.edu.cn

**Keywords:** adaptive signal, A-to-I RNA editing, *Drosophila*, linkage, nonsynonymous

## Abstract

Adenosine-to-inosine (A-to-I) RNA editing leads to a similar effect to A-to-G mutations. RNA editing provides a temporo-spatial flexibility for organisms. Nonsynonymous (Nonsyn) RNA editing in insects is over-represented compared with synonymous (Syn) editing, suggesting adaptive signals of positive selection on Nonsyn editing during evolution. We utilized the brain RNA editome of *Drosophila melanogaster* to systematically study the LD (*r*^2^) between editing sites and infer its impact on the adaptive signals of RNA editing. Pairs of editing sites (PESs) were identified from the transcriptome. For CDS PESs of two consecutive editing sites, their occurrence was significantly biased to type-3 PES (Syn-Nonsyn). The haplotype frequency of type-3 PES exhibited a significantly higher abundance of AG than GA, indicating that the rear Nonsyn site is the driver that promotes the editing of the front Syn site (passenger). The exclusion of passenger Syn sites dramatically amplifies the adaptive signal of Nonsyn RNA editing. Our study for the first time quantitatively demonstrates that the linkage between RNA editing events comes from hitchhiking effects and leads to the underestimation of adaptive signals for Nonsyn editing. Our work provides novel insights for studying the evolutionary significance of RNA editing events.

## 1. Introduction

### 1.1. Adaptive A-to-I RNA Editing in Insects

**Figure 1:**
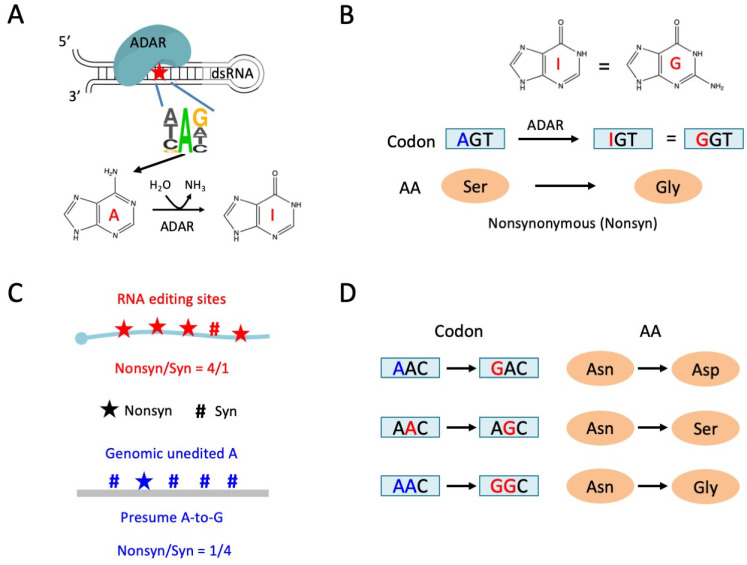
Adenosine-to-inosine (A-to-I) RNA editing and the functional annotation. (**A**) Occurrence of A-to-I RNA editing in metazoans. ADAR acts in *trans* and dsRNA coupled with a 3-mer motif acting in *cis*. (**B**) A-to-I RNA editing in CDS might lead to nonsynonymous mutation. (**C**) Judging the adaptive signals of RNA editome by comparing the observed and expected nonsynonymous/synonymous ratios. Expected nonsynonymous and synonymous sites are obtained by changing all genomic unedited adenosines to guanosines. (**D**) Linkage between RNA editing events will affect the functional annotation of editing sites.

Adenosine-to-inosine (A-to-I) RNA editing is a prevalent type of RNA modification in metazoans [1,2,3]. Adenosine deaminase acting on RNA (ADAR) recognizes double-stranded RNAs (dsRNAs) and catalyzes particular adenosines in the RNA sequences [4,5,6] (Figure 1A). Usually, an A in the HAG motif, where H denotes non-G nucleotides, is prone to being targeted by ADAR [7,8]. ADAR triggers the deamination reaction and converts adenosines to inosines [9]. Due to their base-pairing property, inosines in mRNAs are recognized as guanosines in all cellular processes like reverse transcription and translation [10], and therefore, A-to-I RNA editing has similar consequences to A-to-G mutation [11]. A-to-I RNA editing events in the coding sequence (CDS) are able to cause nonsynonymous changes, altering the protein’s sequence and function (Figure 1B). For example, a nonsynonymous editing (Q > R) in mRNA of the mammalian glutamate receptor GRIA2 is strictly required for survival [12,13,14], suggesting the indispensability of the RNA editing mechanism. In some other model animals, although *ADAR* mutants are viable, they all exhibit neuron-related deficiencies to some extent, such as the defect in chemotaxis observed in *adr-1*/*adr-2*-deleted *Caenorhabditis elegans* [15]. Similarly, *Adar* null mutant of *D. melanogaster* showed retard phenotypes like the lack of locomotion, neurodegeneration, and loss of flight ability [16]. These cases indicate that at least a number of RNA editing sites, especially the nonsynonymous ones, are functional and adaptive. Nevertheless, the total numbers of editing sites vary widely among different species. The human transcriptome contains more than 10^7^ editable adenosine sites, most of which were located in *Alu* repetitive elements [17,18]. In contrast, insects have much fewer editing sites. Several insect species have already been examined; the leaf-cutting ant (*Acromyrmex echinatior*) has ~1.1 × 10^4^ RNA editing sites [7], the bumblebee (*Bombus terrestris*) has ~8.3 × 10^3^ regular editing sites [19], the honeybee (*Apis mellifera*) has ~400 reliable editing sites, and the fruit fly (*D. melanogaster*) has at least ~2 × 10^3^ high-confidence RNA editing sites [20].

However, there are essential differences between A-to-I RNA editing and A-to-G DNA mutation. While DNA mutations are hardwired in the genome, causing potential antagonism between different tissues and developmental stages of organisms (pleiotropic effects), RNA editing provides a temporo-spatial flexibility to control the proteomic diversity, allowing organisms to adapt to changeable environments [21,22,23,24,25,26]. Specifically, the abundance of RNA editing events, together with the expression of *ADAR*, is highest in the nervous systems and brains of animals [27,28,29,30]. It is commonly believed that RNA editing has such an advantage over DNA mutations, and that nonsynonymous RNA editing events are favored by natural selection [31]. The signal of positive selection on nonsynonymous editing sites has been revealed by multiple studies (Figure 1C) [32,33]. Take *Drosophila* for instance, thousands of RNA editing sites were identified in the transcriptome [30,31,34,35]. By comparing the observed nonsynonymous to synonymous ratio (Nonsyn/Syn) of RNA editing sites to the expected Nonsyn/Syn ratio for the numerous unedited adenosines in the genome [36], researchers found that nonsynonymous RNA editing was significantly over-represented in the *Drosophila* transcriptome (Figure 1C) [32], suggesting that these nonsynonymous editing sites were beneficial and were accumulated in the genome during long-term evolution. This is the direct observation of positive selection and thus the adaptive signals on nonsynonymous RNA editing events.

However, the discovery of this adaptive signal requires (1) meticulous identification of RNA editing events to exclude the wide-spread synonymous SNPs (single nucleotide polymorphisms) in the genome, and (2) a strict comparison between the RNA editing sites and the unedited adenosines in the same set of genes. Any technical or methodological biases would introduce false-positive synonymous editing sites that reduce the observed Nonsyn/Syn ratio for RNA editing and dampen the adaptive signals. The confidence in the conclusion of adaptive RNA editing in *Drosophila* still needs to be consolidated by new evidence and data.

### 1.2. Linkage of RNA Editing Events Provides Insights into the Functional Annotation of RNA Editing

In the field of RNA editing, the annotation of editing sites is completely based on the assumption that the editing sites are independent to each other. In bioinformatics, a type of file termed VCF (variant calling format) records each variation site. Each line of VCF contains the functional annotation of one variant. For example, an A-to-G mutation in AAC (1st codon position) will be annotated as Asn > Asp (AAC > GAC), while the A-to-G mutation in AAC (2nd codon position) will be annotated as Asn>Ser (AAC > AGC) (Figure 1D). This annotation is fine if the two variants are far away or within different molecules. However, for the two consecutive A-to-G mutations within the same AAC codon, there is a chance to obtain an Asn > Gly change (AAC > GGC) if the two mutations take place in the same molecule (Figure 1D). The independent annotation of variants will miss such situations. This technical limitation should be more prevalent in RNA editing studies (compared to SNP studies), since RNA editing sites are not randomly distributed and tend to form clusters in the genome [37]. There is an urgent need to unravel how the relationship between different editing sites would affect the function of host genes.

To fill the gap between independent annotation and the potential interaction between RNA editing sites, we previously developed an algorithm to measure the linkage between RNA editing sites [20]. Briefly, we followed the original study that invented the LD (linkage disequilibrium) formula [38] and calculated the pair-wise LD (*r*^2^) and *p* values of each pair of editing sites (PES). The *r*^2^ ranges from 0 to 1 and a higher *r*^2^ represents a stronger LD. We indeed found wide-spread linkage between RNA editing events and proposed that the annotation of editing sites should be updated. However, we did not make further implications on how the linkage between editing events will impact our understanding on the adaptive signals of RNA editing sites.

### 1.3. Aims and Scopes

In this work, we utilized the brain RNA editome of *D. melanogaster* to systematically study the LD (*r*^2^) between editing sites and infer its impact on the adaptive signals of RNA editing. Totally, 1518 PESs were identified. CDS PES had the strongest LD, suggesting potential epistasis between CDS editing sites. For CDS PESs of two consecutive editing sites, including Nonsyn-Nonsyn (type-1), Nonsyn-Syn (type-2), and Syn-Nonsyn (type-3), their occurrence was significantly biased to type-3. The haplotype frequency of type-3 PES exhibited a significantly higher abundance of AG than GA, indicating that the editing of the rear Nonsyn site drives the editing of the front Syn site. Therefore, the Nonsyn sites in type-3 PES act as drivers and the paired Syn sites are passengers. The exclusion of these passenger Syn sites dramatically amplifies the adaptive signal of RNA editing by increasing the observed Nonsyn/Syn ratio for editing sites. Our study for the first time quantitatively demonstrates that the linkage between RNA editing events comes from hitchhiking effects and leads to the underestimation of adaptive signals for Nonsyn editing. Our work provides novel insights for studying the evolutionary significance of RNA editing events.

## 2. Materials and Methods

### 2.1. Data Collection

We retrieved the genome of *D. melanogaster* (Dipteran: Drosophilidae) from FlyBase (https://flybase.org/, accessed on 26 November 2022). The transcriptome (RNA-Seq) data of *Drosophila* brains were downloaded from NCBI (https://www.ncbi.nlm.nih.gov/, accessed on 5 December 2022) under accession SRP074828. The 2114 brain RNA editing sites were downloaded via link (https://doi.org/10.1371/journal.pgen.1006648.s002, accessed on 5 December 2022), and their linkage information was downloaded via link (https://doi.org/10.1093/molbev/msx274, accessed on 5 December 2022). The phyloP score across the *D. melanogaster* genome were downloaded from the UCSC genome browser (http://genome.ucsc.edu/, accessed on 16 January 2023).

### 2.2. LD (Linkage Disequilibrium) Analysis

We followed the pipeline of our previous study [20], which was enlightened by the original literature that described the LD algorithm [38], to calculate the *r*^2^ and *p* values of each pair of RNA editing sites (PES). Due to the limitation of sequencing coverage and read length, only a limited number of RNA editing sites could be covered by the same reads. Not all editing sites in the same gene have a pair-wise LD with each other.

We mapped the RNA-Seq reads to the reference genome using STAR v2.4.2a [39] with default parameters, and 135.6 M reads were mapped. Variations on known RNA editing sites were extracted using sam2tsv (https://github.com/lindenb/jvarkit.git, accessed on 2 June 2023). Consequently, a total of 1518 PESs were identified from the sequencing reads. Notably, each PES should cover at least one editing event. For example, if two nearby editing sites both have 20% editing level, but the “edited reads” at one site did not cover another site (and the reads covering both sites are unedited at both positions), then this pair of editing sites could not be counted as a PES in our results. We only consider the PESs for which we could find a read that contains at least one editing event at one of the two positions. For these PESs, we would count the numbers of the four haplotypes AA, AG, GA, and GG, and the corresponding haplotype frequencies were *f_AA_*, *f_AG_*, *f_GA_*, and *f_GG_*. The calculation of *r*^2^ is based on these haplotype frequencies. The 1518 PESs had *f_AA_* < 1. A PES with *f_AA_* = 1 suggests no editing events were detected at both positions and was not considered as we explained above (but the editing event might be included in other reads that did not cover both positions).

### 2.3. Statistics

The calculation of LD parameters, statistical tests, and graphical works were conducted in Rstudio version 3.6.3.

## 3. Results

### 3.1. Wide-Spread Linkage of RNA Editing Events in the Drosophila Transcriptome

**Figure 2:**
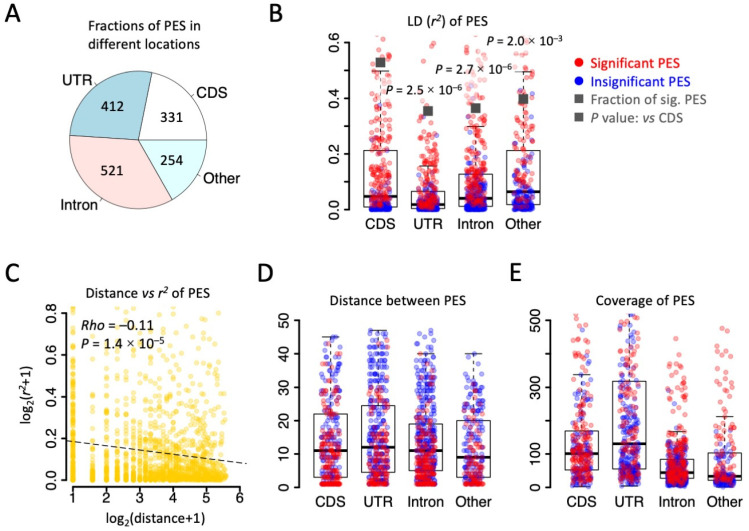
Strong LD observed in the brain transcriptome of *D. melanogaster*. (**A**) Pie chart showing the numbers and fractions of PES in different genomic regions. (**B**) Distribution of *r*^2^ between PES. Significant PESs (*p* < 0.05 in LD) were colored in red. The dark squares represent the fraction of significant PESs in each category. *p* values were obtained by Fisher’s exact tests against the fraction in CDS. (**C**) Spearman correlation between the distance (bp) and *r*^2^ of PESs. All PESs were used. (**D**) Distribution of distance (bp) between PESs in each category. Significant PESs were colored in red. (**E**) Distribution of sequencing coverage on PESs. Significant PESs were colored in red.

We followed the definition of LD [20,38] to calculate the pairwise *r*^2^ between two RNA editing sites that could be covered by the same sequencing reads. With 135.6 M mapped reads from brains of *D. melanogaster*, a total of 1518 pairs of editing sites (PESs) were identified, including 331 PESs in CDS, 412 PESs in UTR, 521 PESs in intron, and 254 PESs in other regions (Figure 2A). The LD (*r*^2^) of CDS PESs was remarkably higher than the *r*^2^ of PESs in UTR and intron (Figure 2B), suggesting stronger linkage between the CDS editing sites compared to non-coding editing events. Accordingly, CDS PESs had the highest fraction of significant PESs across all categories (Figure 2B). Since it is conceivable that the LD (*r*^2^) decreases with distance (Figure 2C), one would predict that CDS editing sites are closer to each other so that they have a stronger LD. However, when we looked at the distance between PESs, no significant differences were seen among different categories (Figure 2D). Moreover, another essential parameter determining the significance of the LD is the sequencing coverage there (resembling the number of alleles in population genetics). Again, we found that the coverage of CDS editing sites was even lower than the coverage of UTR editing sites (Figure 2E). All these results suggest that the strong linkage between CDS editing sites is an intrinsic feature (not caused by technical bias) and should reflect the potential natural selection force on maintaining the linkage between CDS editing events.

Indeed, it is possible that the CDS RNA editing sites, especially the nonsynonymous ones, have epistatic effects, and that the maintenance of such linkage between these editing sites comes at the cost of a reduced genome evolution rate. Then, we focused on CDS editing sites and investigated how the linkage between editing sites affects the distribution of nonsynonymous and synonymous editing sites.

### 3.2. Biased Composition of Adjacent PESs Suggests Potential Interaction between Editing Sites

**Figure 3:**
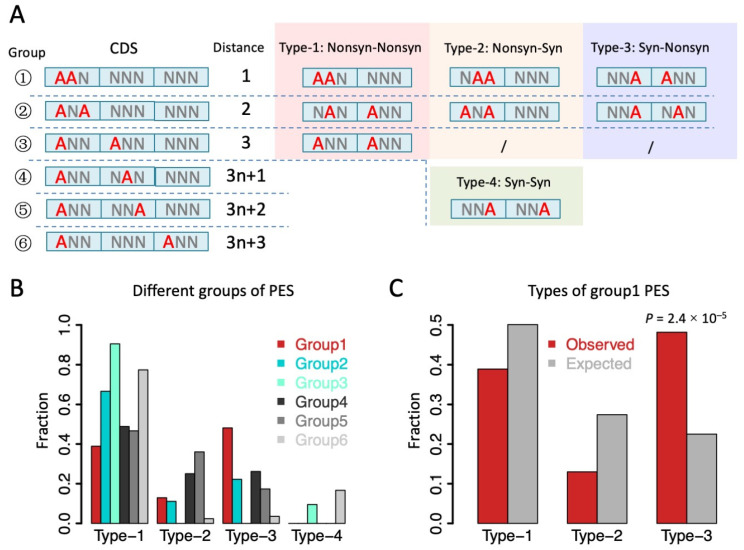
Classification of PESs in CDS according to the distance between two editing sites and their annotation. (**A**) Definition of different types of PES with different distances. For simplicity, A-to-I(G) editing at the 3rd codon position was treated as a synonymous event, despite there being one exception. Red “A”s indicate RNA editing sites. (**B**) Observed fractions of each type of PESs in different groups. Type-3 PES was obviously over-represented in group1 PESs. (**C**) Observed and expected fractions of three types of PESs for group1 (two adjacent editing sites). *p* value was calculated by Chi-square test.

When interrogating the relationship between two RNA editing sites (e.g., PES), an essential difference between CDS PESs and non-coding PESs is the existence of tri-nucleotide periodicity (reading-frame) in CDS. For PESs in non-coding regions, the distance between two sites has no direct effect on the functional consequence of the two editing sites. However, in CDS, A-to-I(G) RNA editing at the 1st and 2nd codon positions will leads to nonsynonymous (Nonsyn) mutations and the editing at the 3rd codon position leads to synonymous (Syn) mutations, except for one case (ATA > ATG, Ile > Met). For PESs in CDS, the distance between two editing sites, together with the frame of the first site, will determine whether the two sites are annotated as type-1 (Nonsyn-Nonsyn), type-2 (Nonsyn-Syn), type-3 (Syn-Nonsyn), or type-4 (Syn-Syn) (Figure 3A). First, we classified all CDS PESs into six groups according to the distance (bp) between them: PESs with d = 1, 2, 3, 3n + 1, 3n + 2, and 3n + 3 (n > 0) were defined as groups 1~6, respectively. Then, within each group, we further classified the PESs into the four types according to the annotation (Figure 3A).

We found that for most groups of PESs, the numbers of type-1 PES (Nonsyn-Nonsyn) were much higher than the numbers of other types of PES (types-2, 3, 4 that contain a Syn site) (Figure 3B). This supports the notion that there are epistatic effects between Nonsyn editing sites. Surprisingly, only in group1 PESs (distance = 1 bp), we found a remarkably high fraction of type-3 PESs (Syn-Nonsyn) (Figure 3B). The numbers and fractions of types 1, 2, and 3 were 21 (38.9%), 7 (13.0%), and 26 (48.1%) for group1 PESs, and this composition was obviously biased towards type-3 PESs compared with the profile in groups 2–6 (Figure 3B). We argue that this excess of type-3 PESs in group1 is not caused by the constraint of the reading-frame because the group4 PESs with distance = 3n + 1 (which had the same frame with group1) did not show a high proportion of type-3 PESs (Figure 3B). Therefore, the only plausible trigger of this pattern is the fact that the two sites of group1 PESs are closely adjacent to each other.

To test how unexpected it is to see such a high proportion of type-3 PESs among group1, we needed to find a negative control. We calculated the numbers of two consecutive (unedited) adenosines in the CDS regions of the *D. melanogaster* genome. Using the genome-wide data as a control, we found that the expected numbers and fractions of types 1, 2, and 3 were 754,666 (50.1%), 412,912 (27.4%), and 338,895 (22.5%) (Figure 3C), respectively. These fractions were significantly different from the observed numbers in group1 PESs (Figure 3C, *p* = 2.4 × 10^−5^, Chi-square test). The over-represented type-3 (Syn-Nonsyn) PESs in group1 might reflect a non-random process between two editing sites. Next, we investigated the group1 PESs and studied the possible interactions between two neighboring editing sites.

### 3.3. Haplotype Frequency Suggests Many Synonymous Editing Sites Are Passengers

**Figure 4:**
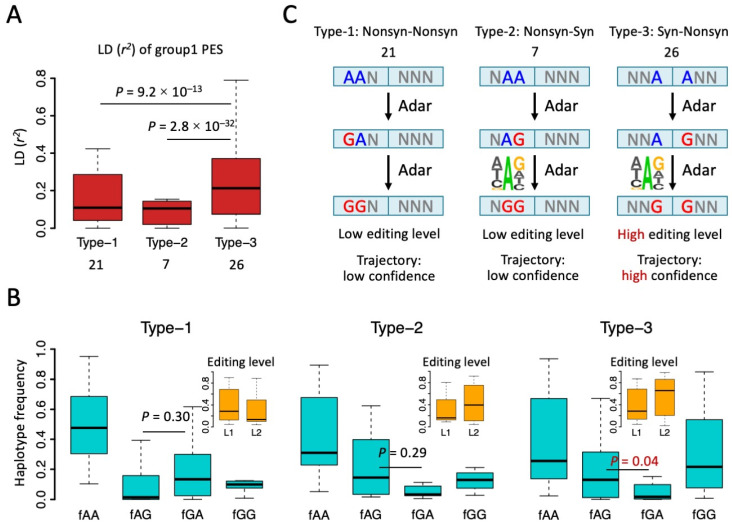
Type 1, 2, and 3 of group1 PESs and the trajectory of editing process. (**A**) Boxplot showing the *r*^2^ of group1 PESs. The numbers of each type of PES were shown below. *p* values were calculated by Wilcoxon rank sum tests under 1000 times bootstrap. (**B**) Haplotype frequencies (blue) of the four combinations AA, AG, GA, and GG of PESs. Types 1, 2, and 3 were displayed separately. Editing levels of two sites (L1 and L2) were displayed in the small panel. *p* values were calculated between *f_AG_* and *f_GA_* using paired Wilcoxon rank sum tests. (**C**) Putative trajectory of editing process inferred from haplotype frequencies. Unedited “A”s are in blue and edited sites are in red.

Since we noticed that only group1 PESs (two adjacent editing sites) had an extraordinarily high fraction of type-3 PESs (Syn-Nonsyn), we set out to explain this unique phenomenon. We first looked at the strength of LD (*r*^2^) of those PESs (Figure 4A). Type-3 PESs had a significantly higher *r*^2^ than the other two types of PES (Figure 4A). This echoes the over-representation of type-3 PESs and suggests an intrinsic mechanism promoting the co-occurrence of these editing events. To better understand the editing process on PESs, we calculated the haplotype frequencies of the four combinations. For all the three types of group1 PESs, AA had the highest haplotype frequency (Figure 4B). This agrees with the fact that most CDS editing sites in *Drosophila* had a level lower than 50%, usually with a median value around 20% [28,31,33,40,41]. Then, we looked at the haplotype frequencies of the single edited haplotypes AG and GA (Figure 4B). AG means the second (rear) site is edited and GA means the first (front) site is edited. Interestingly, for type-1 PESs, GA was more abundant than the AG haplotype, while for type-2 and type-3 PESs, AG is more abundant than GA (Figure 4B). However, this difference between *f_AG_* and *f_GA_* was only significant for type-3 PESs (Figure 4B).

The relative abundance of AG and GA will imply the potential trajectory of how these two editing sites were edited. For example, for type-2 and type-3 PESs, it is very likely that the rear editing site was edited at first, providing a favorable context for the front site, and then the front site was edited (Figure 4C). Notably, in metazoans, the favorable sequence context for RNA editing site is mainly determined by the 3-mer motif surrounding the focal editing site, where the upstream nucleotide avoids G and the downstream nucleotide favors G [7,8]. For type-2 and type-3 PESs, editing at the rear site creates an AG context for the front editing site, increasing the probability that the front site will be edited (Figure 4C). For type-1 PESs, since the GA haplotype is more abundant than the AG haplotype (Figure 4B), the most likely process is AA-to-GA-to-GG (Figure 4C). This seems to contradict the known editing preference at site 2. However, we would explain this dilemma in two different ways. (1) *f_AG_* and *f_GA_* was only significantly different for type-3 PESs (Figure 4B), suggesting that the editing trajectories inferred from the haplotype frequencies might be unreliable for type-1 and type-2 PESs (Figure 4C); (2) When we measured the editing level at two sites (where level_1_ = [GA + GG]/[GA + AG + GG + AA] and level_2_ = [AG + GG]/[GA + AG + GG + AA]), we could clearly see that the editing levels in type-1 and type-2 PESs were much lower than the editing levels in type-3 PESs because the GG haplotype had a high frequency only in type-3 PESs (Figure 4B,C). For example, the editing level of site2 in type-3 PESs was as high as 70% (median), while the median editing levels for sites in type-1 and type-2 PESs were no higher than 30% (Figure 4B). This suggests that editing sites in type-3 PESs are functionally more important than the sites in the other two types of PES, or the linkage event itself is more important for type-3 PESs compared to type-1 and type-2 PESs. Therefore, we only focus on type-3 PESs in the following analyses. The seemingly unreasonable editing trajectory of type-1 PESs only accounts for a small fraction of less essential sites.

### 3.4. Underestimation of Adaptive Signals of RNA Editing Due to the Hitchhiking of Synonymous Sites

Interestingly, according to the inferred editing process of type-3 PESs (Figure 4C), the rear Nonsyn site should be the driver, which is the main target of RNA editing, and the front Syn site should be the passenger, which is the byproduct of the editing of the driver site. Importantly, a key criterion to judge the evolutionarily adaptive signal of A-to-I RNA editing is the comparison between the observed Nonsyn/Syn ratio and the random (neutral) expectation if one changes all adenosines to guanosines in the reference genome [32,42].

**Figure 5:**
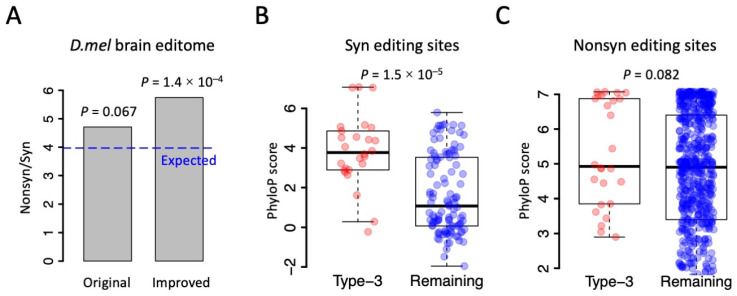
Adaptive signal of RNA editing is frequently affected by the linkage between editing sites. (**A**) The observed and expected ratios of Nonsyn/Syn. *p* values were calculated using Fisher’s exact tests. (**B**) PhyloP scores of Syn editing sites. *p* value was calculated using Wilcoxon rank sum test. (**C**) PhyloP scores of Nonsyn editing sites. *p* value was calculated using Wilcoxon rank sum test.

The *Drosophila* brain editome recorded 678 Nonsyn editing sites and 144 Syn editing sites. The Nonsyn/Syn ratio is 4.71 (Figure 5A), which is remarkably higher than the random expectation at the genome-wide level (Nonsyn/Syn = 11,862,949/2,988,735 = 3.97). This difference is marginally significant (Figure 5A, *p* = 0.067 by Fisher’s exact test); although, the expected Nonsyn/Syn could be slightly reduced when only the edited genes were considered. Here, if the 26 passenger Syn editing sites in type-3 PESs were removed, the observed Nonsyn/Syn ratio will be 678/118 = 5.75, which is significantly higher than the random expectation of 3.97 (Figure 5A, *p* = 1.40 × 10^−4^), regardless of whether the edited genes or all genes were used. Therefore, studying the linkage between CDS editing sites would help us to clarify which editing sites are the main target of natural selection and which sites are just byproducts. This information would also deepen our understanding of the adaptive nature of the RNA editing mechanism in *Drosophila* as well as other insects and metazoans.

### 3.5. Synonymous Sites in Type-3 PESs Were More Conserved than Other Synonymous Editing Sites

Based on the fact that many of the Syn editing sites in the *Drosophila* editome come from hitchhiking, which means that they are byproducts of Nonsyn editing events, we wondered whether we could find more evidence and data to enlarge the adaptive signals of RNA editing events. Auxiliary evidence for adaptive editing is that the Nonsyn editing sites are usually (genomically) more conserved than the Syn editing sites [8,31]. For the fruit fly *D. melanogaster*, the conservation level of each genomic site could be quantitatively measured using the phyloP score (http://genome.ucsc.edu/, accessed on 1 september 2023). A higher phyloP score represents a higher conservation level of a site across the phylogeny. We found that the 26 Syn editing sites in type-3 PESs had significantly higher conservation levels than the remaining Syn editing sites (Figure 5B). In contrast, the 26 Nonsyn editing sites in type-3 PESs did not show a significant difference in the conservation level with the remaining Nonsyn editing sites (Figure 5C). Since the Syn editing sites in type-3 PESs were passengers that should not be counted as the “real Adar targets”, the remaining Syn editing sites (which had lower conservation levels) would be even less conserved than the Nonsyn editing sites. This enlarges the adaptive signals by amplifying the differential conservation levels between Nonsyn and Syn editing sites. This interesting observation could not be discovered without considering the linkage information between RNA editing sites.

## 4. Discussion

Whether and how nonsynonymous RNA editing events are evolutionarily adaptive remains debatable, but this debate mainly converges to the cephalopods, which have incredibly abundant nonsynonymous editing events [43,44]. For other clades, the evolutionary significance of RNA editing is quite clear. In insects like *Drosophila* and honeybees, nonsynonymous RNA editing diversifies the proteome in a temporo-spatial manner, and this mechanism provides flexibility for the organisms to adapt to a changeable environment [36]. The same purpose of RNA editing has been proposed in fungi [22]. In mammals, the majority of nonsynonymous editing sites came from promiscuous targeting of ADARs and did not increase the fitness of hosts [42,45]. In vascular plants, it is almost a consensus that RNA editing is used for reversing deleterious DNA mutations and then restoring the ancestral allele [41]. Therefore, our study is not designed to resolve the debate on adaptive editing. Instead, based on the consensus that nonsynonymous RNA editing in *Drosophila* is beneficial due to the proteomic diversifying role, we tried to consolidate this notion by showing that the currently observed adaptive signal has even been underestimated.

By systematic identification of the linked RNA editing sites in the *Drosophila* brain transcriptome, we found a particular class of editing pairs (what we called type-3 PES of group1) that showed strong linkage and a biased profile of haplotype frequency. We argue that the synonymous editing site within this PES is the passenger produced by the editing of adjacent nonsynonymous site. Then, these passenger synonymous sites should be excluded in the evolutionary analyses involving Nonsyn/Syn ratios. This is a key conclusion based on our observations.

The measurement of linkage between RNA editing sites is a novel field with technical challenges. The idea came from the LD measurement in population genetics [20,38]. Within a population, the phase of SNPs could be inferred from multiple features so that the linkage map of SNPs could be extended to distantly located regions. For homozygous SNPs in an individual, their linkage would be 100% regardless of the recombination events. However, for RNA editing sites, the detection of linkage completely relies on the sequencing reads covering multiple editing sites. This inevitable limitation largely reduces the detectable distance between editing sites. The pair-ended 150 bp sequencing might cover multiple editing sites within several hundred bps, but this maximum distance is still insufficient to meet the demands for building the entire linkage map of all RNA editing sites in the transcriptome. Promisingly, methodologies for identifying RNA editing sites from the third-generation sequencing data are emerging [46], and this breakthrough might shed light on our understanding of the complete linkage map of RNA editing sites across the transcripts. At this stage, to avoid the limitation of read length, we only focused on the adjacent RNA editing sites (group1) and investigated their editing trajectory.

Notably, in our analyses, although we observed that the LD (*r*^2^) between RNA editing sites decreases with distance, which is similar to what should be observed for DNA mutations, the mechanisms are largely different. The linkages between DNA mutations are eroded by the recombination of chromatids: the farther apart two mutations are located, the higher probability they will be separated by recombination. For RNA editing sites, the linkage between two closely related sites is simply caused by the “batch production” of ADAR proteins: the farther apart the two editing sites are located, the less likely they will be edited by ADARs at the same time. This is the essential difference between RNA editing and DNA mutation.

Taken together, we found that the CDS editing sites are strongly linked to each other. For the two consecutive editing sites spanning two codons, the rear Nonsyn site is the driver that promotes the editing of the front Syn site (passenger). The exclusion of passenger Syn sites dramatically amplifies the adaptive signal of Nonsyn RNA editing (by elevating the Nonsyn/Syn ratio). The linkage information should be considered when studying the functional consequence and evolutionary significance of RNA editing sites.

## 5. Conclusions

Our study for the first time quantitatively demonstrates that the linkage between RNA editing events comes from hitchhiking effects and leads to the underestimation of adaptive signals for Nonsyn editing. Our work provides novel insights for studying the evolutionary significance of RNA editing events.

## Data Availability

The genome of *D. melanogaster* (Dipteran: Drosophilidae) was downloaded from FlyBase (https://flybase.org/, accessed on 26 November 2022). The transcriptome (RNA-Seq) data of *Drosophila* brains were downloaded from NCBI (https://www.ncbi.nlm.nih.gov/, accessed on 5 December 2022) under accession SRP074828. The 2114 brain RNA editing sites were downloaded via link (https://doi.org/10.1371/journal.pgen.1006648.s002, accessed on 5 December 2022), and their linkage information was downloaded via link (https://doi.org/10.1093/molbev/msx274, accessed on 5 December 2022). The phyloP score across the *D. melanogaster* genome were downloaded from the UCSC genome browser (http://genome.ucsc.edu/, accessed on 16 January 2023).

## Abbreviations

ADAR	adenosine deaminase acting on RNA
A-to-I	adenosine-to-inosine
CDS	coding sequence
LD	linkage disequilibrium
Nonsyn	nonsynonymous
PES	pair of editing sites
Syn	synonymous
VCF	variant calling format
